# Elicitation of Highly Pathogenic Avian Influenza H5N1 M2e and HA2-Specific Humoral and Cell-Mediated Immune Response in Chicken Following Immunization With Recombinant M2e–HA2 Fusion Protein

**DOI:** 10.3389/fvets.2020.571999

**Published:** 2021-02-05

**Authors:** Semmannan Kalaiyarasu, Sandeep Bhatia, Niranjan Mishra, Dhanapal Senthil Kumar, Manoj Kumar, Richa Sood, Katherukamem Rajukumar, Boopathi Ponnusamy, Dhruv Desai, Vijendra Pal Singh

**Affiliations:** ^1^Indian Council of Agricultural Research-National Institute of High Security Animal Diseases, Bhopal, India; ^2^Indian Council of Agricultural Research-Indian Veterinary Research Institute, Bareilly, India

**Keywords:** avian influenza, immunity, matrix and hemagglutinin, humoral and cell mediated immunity, recombinant protein

## Abstract

The study was aimed to evaluate the elicitation of highly pathogenic avian influenza (HPAI) virus (AIV) M2e and HA2-specific immunity in chicken to develop broad protective influenza vaccine against HPAI H5N1. Based on the analysis of Indian AIV H5N1 sequences, the conserved regions of extracellular domain of M2 protein (M2e) and HA2 were identified. Synthetic gene construct coding for M2e and two immunodominant HA2 conserved regions was designed and synthesized after codon optimization. The fusion recombinant protein (~38 kDa) was expressed in a prokaryotic system and characterized by Western blotting with anti-His antibody and anti-AIV polyclonal chicken serum. The M2e–HA2 fusion protein was found to be highly reactive with known AIV-positive and -negative chicken sera by ELISA. Two groups of specific pathogen-free (SPF) chickens were immunized (i/m) with M2e synthetic peptide and M2e–HA2 recombinant protein along with one control group with booster on the 14th day and 28th day with the same dose and route. Pre-immunization sera and whole blood were collected on day 0 followed by 3, 7, 14, 21, and 28 days and 2 weeks after the second booster (42 day). Lymphocyte proliferation assay by 3-[4,5-dimethylthiazol-2-yl]-2,5 diphenyl tetrazolium bromide (MTT) method revealed that the stimulation index (SI) was increased gradually from days 0 to 14 in the immunized group (*p* < 0.05) than that in control chicken. Toll-like receptor (TLR) mRNA analysis by RT-qPCR showed maximum upregulation in the M2e–HA2-vaccinated group compared to M2e- and sham-vaccinated groups. M2e–HA2 recombinant protein-based indirect ELISA revealed that M2e–HA2 recombinant fusion protein has induced strong M2e and HA2-specific antibody responses from 7 days post-primary immunization, and then the titer gradually increased after booster dose. Similarly, M2e peptide ELISA revealed that M2e–HA2 recombinant fusion protein elicited M2e-specific antibody from day 14 onward. In contrast, no antibody response was detected in the chicken immunized with synthetic peptide M2e alone or control group. Findings of this study will be very useful in future development of broad protective H5N1 influenza vaccine targeting M2e and HA2.

## Introduction

Influenza A viruses have been isolated from a wide range of animals including poultry, wild and cage birds, pigs, horses, dogs, sea mammals, and humans, although ducks are considered the natural reservoirs of avian influenza viruses (AIVs). Based on the antigenicity of two viral glycoproteins *viz* hemagglutinin (HA) and neuraminidase (NA), influenza A viruses are further classified into subtypes; to date, though 18 HA subtypes (H1–H18) and 11 NA subtypes (N1–N11) have been identified (1), only 16 HA subtypes (H1–H16) and nine NA subtypes are considered true influenza viruses and the remaining two, namely, H17N10 (2) and H18N11 (3), are considered influenza A-like viruses (1). In South Asia, the H5N1 virus was first reported in domestic poultry in India and Pakistan during February 2006 and followed by Bangladesh, Nepal, and Bhutan in March 2007, January 2009, and February 2010, respectively (4). All the H5N1 viruses isolated from poultry and humans in South Asia until 2010 belong to clade 2.2 (5–8). The first introduction of clade 2.3.2 H5N1 virus to South Asia was reported from Nepal in February 2010 (9, 10), followed by in India in February 2011 (11). Antigenic analysis showed 64–256-fold reduction of cross reactivity in clade 2.3.2.1 as compared to clade 2.2 viruses, which revealed that the likelihood of clade 2.2 viruses to provide cross-protection against 2.3.2.1 viruses is less (11). Between November 2014 to March 2015, clade 2.3.2.1c has been reported as the new introduction to India (12), followed by worldwide circulation of clade 2.3.4.4 including India (13–17). Due to the continuous change of clades, cross protection between the clades become uncertain.

M2 is a type III integral membrane protein forming a pH-dependent proton-selective ion channel (18, 19) produced by spliced mRNA translation of gene segment 7 of influenza virus, which also codes for M1 protein (20). The M2 protein (96 amino acids) contains three structural domains, namely, amino-terminal extracellular domain M2e (23 residues), a transmembrane domain (19 residues), and a cytoplasmic domain (54 residues) (21), which gives the native tetrameric conformation with disulfide bonds. Though M2 protein molecules are estimated to be present at low level (20–60 numbers) on each virion, they are expressed at high levels on the surface of infected cells (22). The amino acid sequence in M2e is highly conserved among influenza A viruses (23, 24). The five amino acids within the residues 10–20 of M2e were observed to be host restricted: PIRNEWGCRCN (amino acids 10–20, human isolates), PTRNGWECKCS (amino acids 10–20, avian isolates), and PIRNGWECRCN (amino acids 10–20, swine isolates) (24). Due to the low degree of variation in the M2 extracellular domain, it is considered an attractive antigenic target for developing a universal influenza vaccine.

Influenza virus HA is a homotrimeric protein molecule, and each monomer consists of two disulfide-linked subunit glycoproteins, a globular head of HA1 and a stem or stalk domain composed of the N- and C-terminal parts of HA1 and all of HA2 (25). HA is synthesized as a precursor (HA0) that is cleaved into HA1 and HA2 domains. The cleavage site of HA with the fusion peptide and N-terminal portion of HA2 is the most conserved sequence among influenza A viruses and has the potential application as a universal antigen. Although HA stem region is considered a good option for the development of the universal vaccine, the frequency of anti-stem antibodies is considerably lower than that of anti-globular head antibodies in natural infection (26) due to the physical masking of immunodominant head over the stem region and close proximity of stem epitope(s) to the viral membrane (27).

It is always advantageous to add more conserved immunogenic regions to get better cross-protection while developing a universal vaccine instead of selecting a single region. Most of the studies had been carried out to assess the immunogenicity of M2e and HA2 region of stalk domain in either mice or pigs. But it is essential to evaluate these types of conserved region-based immunogens in chicken before applying the universal vaccine strategy in poultry industry against H5N1 or other highly pathogenic AIV infections. Hence, the study was aimed to develop highly pathogenic avian influenza (HPAI) virus M2e and HA2-specific immunity in chicken to develop a broad protective influenza vaccine against HPAI H5N1.

## Materials and Methods

### Identification of M2e and HA2 Conserved Region of Indian Avian Influenza H5N1 Viruses

For the identification of M2e and HA2, all the clades of Indian AIV H5N1 virus M2 and HA sequences of 2006 to 2015 outbreaks were included and analyzed by MegAlign software (DNASTAR, Inc., USA). Conserved regions of extracellular domain of M2 protein (M2e) and HA2 were identified, and the identity was compared with published M2e and HA2 sequences.

### Synthesis of Avian Influenza M2e Antigen

The identified M2e (2–24 amino acid) (SLLTEVETPTRNEWECRCSDSSD) was synthesized commercially (Genscript, USA) as a synthetic peptide antigen.

### Expression and Characterization of M2e–HA2 Fusion Recombinant Protein

**Figure 1:**
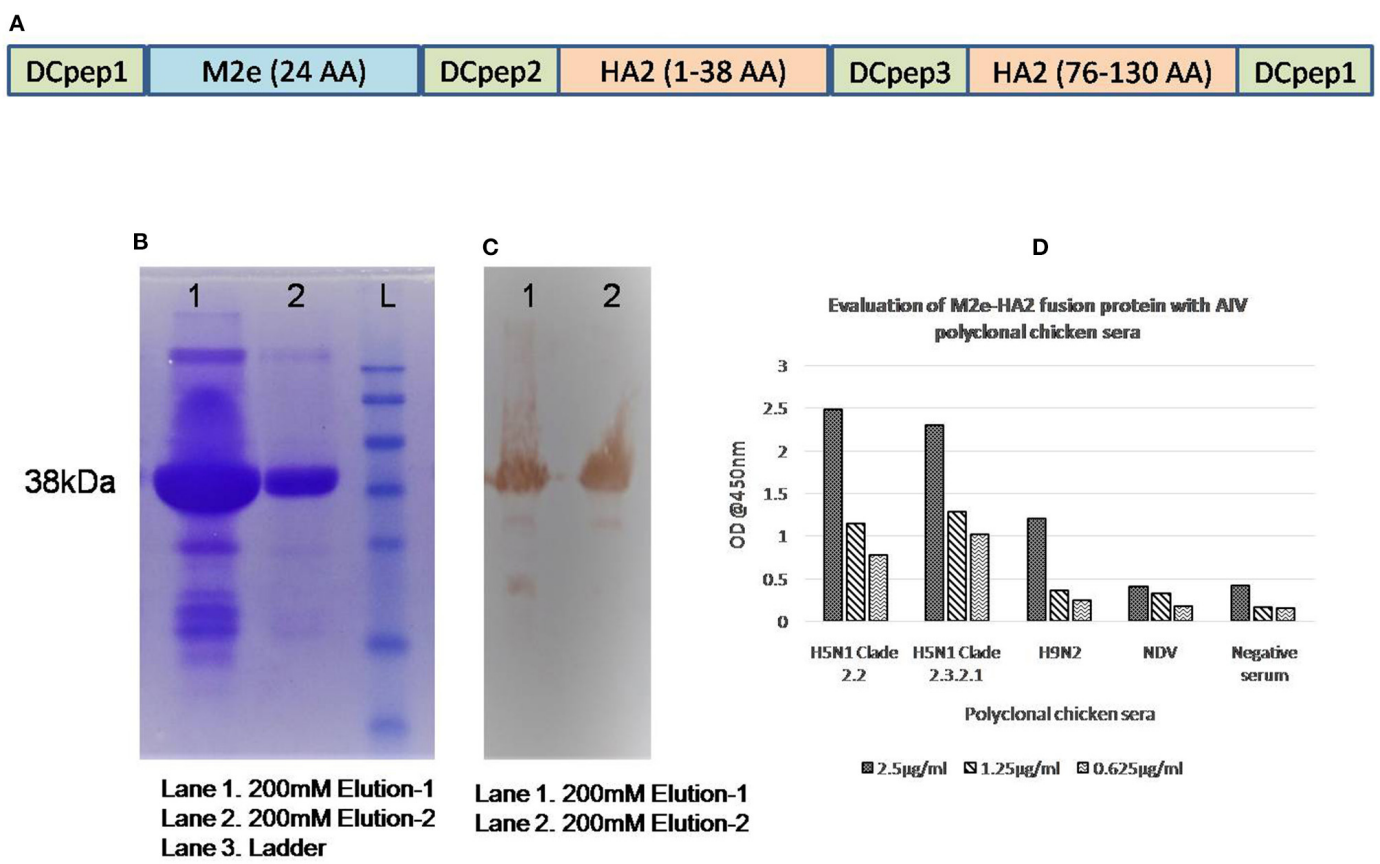
Gene construction and expression of M2e–HA2 recombinant protein in Escherichia coli Rosetta Blue (*DE3*)*pLysS*. Sodium dodecyl sulfate-polyacrylamide gel electrophoresis (SDS-PAGE) and Western blot analysis and evaluation of recombinant M2e–HA2 protein by indirect ELISA with positive control sera. **(A)** Synthetic gene constructs coding for M2e (2–24 AA) and HA2 conserved regions (N-terminal 1–38 AA and Long α-helix 76–130 AA) with chicken dendritic cell binding peptides were designed by placing glycine linkers (GGG) in between them. **(B)** SDS-PAGE analysis of recombinant M2e–HA2 protein by Coomassie blue stain and **(C)** Western blot analysis of recombinant M2e–HA2 protein (38 kDa) showed high reactivity with polyclonal avian influenza virus (AIV) serum. **(D)** Indirect ELISA with recombinant M2e–HA2 fusion protein showed high reactivity with different clades (2.2 and 2.3.2.1) of H5N1 AIV, H9N2 AIV but failed to react with NDV and control chicken serum.

Synthetic gene constructs coding for M2e (2–24 AA) and HA2 conserved region (N-terminal 1–38 AA and Long α-helix 76–130 AA) were designed by placing glycine linkers (GGG) and chicken dendritic cell binding peptides (amino acid sequences of chicken dendritic cell binding peptides were not shown) in between them and synthesized commercially after codon optimization (Genscript, NJ, USA) in pET 32b(+) vector system along with His-Tag. Then, the gene was used for the expression of recombinant protein in BL21 (DE3) pLysS cells and purified using His-Bind purification kit (Merck Millipore, USA). Briefly, a single colony of transformed Escherichia coli Rosetta Blue (*DE3*)*pLysS* was incubated overnight on a shaker incubator in 2 ml LB medium containing ampicillin (100 μg/ml) and chloramphenicol (34 μg/ml) at 37°C with constant agitation (200 rpm). The next day, 500 μl of culture was inoculated in 50 ml LB broth (1/100) and grown up to an OD_600_ of 0.6 with vigorous shaking (200 rpm) at 37°C. Isopropyl-β-D-thiogalactopyranoside (IPTG) was added to a final concentration of 1 mM for expression of fusion protein in E. coli and incubated further for another 4 h at 37°C with shaking at 200 rpm. In order to produce the expression protein, bacterial suspensions were tested at 2- and 4-h intervals and analyzed on 12% sodium dodecyl sulfate (SDS)-polyacrylamide gel electrophoresis (PAGE). The fusion chimeric protein (M2e–HA2) in the induced cell pellet as inclusion bodies was purified using the BugBuster® HisBind® Purification Kit (Novagen, USA) following the manufacturer's protocol. The purified M2e–HA2 recombinant protein was characterized by Western blotting with anti-His antibody and anti-AIV polyclonal chicken serum (Figure 1). Similarly, reactivity of M2e–HA2 fusion protein with known AIV-positive and -negative chicken sera was tested by ELISA (Figure 1D).

### Preparation of Immunogen

#### M2e Synthetic Peptide and M2e–HA2 Fusion Recombinant Protein Emulsification

Six milliliters of synthetic peptides (M2e) and recombinant protein (M2e–HA2) were emulsified with Montanide^TM^ ISA 71 VG (SEPPIC) (14 ml) in the ratio of 3:7 to prepare water-in-oil (W/O) emulsion. Sham vaccine for control birds was prepared by emulsifying phosphate buffered saline (PBS) with Montanide^TM^ ISA 71 VG.

#### Immunization of Specific Pathogen-Free Chickens With the Water-In-Oil Emulsified Synthetic Peptide (M2e) and Recombinant Protein (M2e–HA2) Antigen

Four-week-old specific pathogen-free (SPF) chickens were immunized with 0.5 ml of emulsified antigen containing 100 μg of peptide (M2e) or 100 μg of recombinant protein (M2e–HA2) per dose in breast muscle. Six birds in each group were immunized with M2e synthetic peptide (A), recombinant M2e–HA2 fusion protein (B), and six birds were sham vaccinated (C) apart from unvaccinated control birds (D). Booster was given on the 14th day and 28th day with the same dose and route. Pre-immunization sera and whole blood with ethylenediaminetetraacetic acid (EDTA) were collected on day 0, followed by 3, 7, 14, 21, and 28 days, and 2 weeks after the second booster (42nd day). All the samples were processed on the same day of collection.

### Measurement of Cell-Mediated Response

#### Separation of Peripheral Blood Mononuclear Cells From Chicken Blood

Approximately 3 ml of peripheral blood was collected from the wing vein of each bird following sterile procedure and immediately transferred into tubes containing EDTA. Buffy coat (750 μl) was separated by centrifugation at 1,800 rpm for 40 min. A 2-ml microcentrifuge tube, 750 μl of Histopaque®-1077 was taken, and 750 μl of buffy coat was gently layered over it. Centrifugation for 30 min at 1,800 rpm was done in room temperature. The mononuclear cells were aspirated from the opaque interface of the upper layer. The cells were washed thrice with sterile PBS followed once with RPMI 1640 medium by centrifuging at 1,000 rpm for 10 min. Cells were resuspended in 1 ml of RPMI 1640 and counted using Neubauer chamber. Then, the peripheral blood mononuclear cells (PBMCs) were made into different aliquots to carryout FACS, lymphocyte proliferation assay, and cytokine and Toll-like receptor (TLR) mRNA expression studies.

#### Flow Cytometry Analysis of Chicken Peripheral Blood Mononuclear Cells

PBMCs of post-immunized chicken were processed for the analysis with flow cytometry using anti-chicken CD4 and CD8 fluorescein isothiocyanate (FITC) MAbs (Southern Biotech, USA). Briefly, 100 μl of PBMCs (10^5^-10^6^ cells) in PBS was mixed with 5 μl of MAbs (0.5 μg/μl) in individual tubes each along with an isotype control for individual birds. Cells were mixed by gentle vortexing and incubated at 37°C for 1 h. Then, the tubes were washed thrice with washing *cum* blocking buffer containing PBS, 1% bovine serum albumin (BSA), and 0.1% sodium azide (SA) by centrifugation at 4,000 rpm for 5 min. Then, the cells were resuspended and fixed with 0.5% paraformaldehyde (PFA) for 30 min at room temperature and analyzed by flow cytometry FACSCanto (BD Biosciences, San Jose, CA, USA). The results were analyzed with FACSDiVa® software (BD Biosciences, San Jose, CA, USA).

#### Lymphocyte Proliferation Assay

Lymphocyte proliferation assay was performed as described previously (28) using CellTiter 96® Non-Radioactive Cell Proliferation Assay kit (Promega, Madison, WI). Briefly, triplicates of 1–2 × 10^5^ number of PBMCs from each bird of different groups were cultured in 96-well plates in 100 μl of RPMI 1640 medium. Ten micrograms of M2e peptide or recombinant protein (M2e-HA2) were used as stimulating antigen in their corresponding groups and concanavalin A (ConA) and lipopolysaccharide (LPS) as positive controls in triplicate wells for each sample. Similarly, triplicate wells of each bird were kept as unstimulated control within the group. The cells were incubated in a final volume of 100 μl complete RPMI 1640 for 72 h, and 15 μl of dye solution [3-[4,5-dimethylthiazol-2-yl]-2,5 diphenyl tetrazolium bromide (MTT)] was added to the plates and incubated further for 4 h at 37°C. Then, 100 μl of solubilization solution was added to all the wells. Then, the plates were kept on an orbital shaker for 10 min, and the absorbance was finally measured at 550 nm using TriStar2S LB 942 multimode reader (BERTHOLD Technologies, Germany). The proliferation index or stimulation index (SI) was calculated compared to negative control, and the results were expressed as the mean of triplicate wells. The proliferation index was calculated by the following formula:

Mean OD_550_ of antigen-treated well—Mean OD_550_ of blank/Mean OD_550_ of unstimulated control well.

### Quantification of Cytokines and Toll-Like Receptors mRNA Expression in Chicken Peripheral Blood Mononuclear Cells

The mRNA expressions of chicken cytokines [transforming growth factor (TGF)-β, tumor necrosis factor (TNF)-α, interferon (IFN)-α, IFN-β, IFN-γ, interleukin (IL)-1β, IL-6, IL-4, and IL-10] and TLRs (1, 2, 3, 4, 5, 7, 15, and 21) were quantified using primers listed in our earlier work (29, 30) by RT-qPCR using Light Cycler 480 SYBR Green I master (Roche, Germany) in Light Cycler® 480 Real Time PCR System II (Roche, Germany). Total RNA was extracted from PBMCs using RNeasy minikit (Qiagen, USA) according to the manufacturer's protocol, and cDNA synthesis was carried out using First Strand cDNA Synthesis Kit (Fermentas Life Sciences, USA) with random hexamer primer and SuperScript II Reverse Transcriptase from 1 μg of RNA. Then, SYBR green-based qPCR was performed as per the instructions of the manufacturer. Briefly, A total reaction volume of 20 μl containing 10 μl of 2× SYBR Green I master mix, 2 μl of cDNA, 1 μl of primers each (20 pmol) was used for amplification in triplicates with the following thermal profile: one cycle of 95°C for 2 min, 40 cycles of 95°C for 10 s, 60°C for 30 s, and 72°C for 30 s. The fluorescence was measured for every cycle at the end of extension, and amplification product dissociation was analyzed at the end of the PCR. Chicken β-actin gene from the same sample was used as a reference gene for normalization. Means of triplicate reactions were used to determine mean cycle threshold (Ct) value of three birds (*n* = 3, data points = 9), respectively. Comparative Ct value was used to determine fold changes in gene expression, calculated as 2^−Δ*ΔCt*^ (31) by using Relative Expression Software Tool (REST) 2009. Data were analyzed using REST 2009, means and SE were calculated using REST software, i.e., the results of the 2,000 random reallocations. The software uses pairwise fixed reallocation randomization test to calculate the *p*-values between groups. *p* < 0.05 was considered statistically significant.

### Assessment of Humoral Immune Response by M2e Synthetic Peptide, HA Stalk Recombinant Protein, and M2e–HA2 Recombinant Protein-Based Indirect ELISA

Experimental hyper immune chicken sera of different clades were tested by ELISA using M2e synthetic peptide and M2e–HA2 recombinant fusion protein by indirect ELISA to ensure its reactivity (32). Sera from all the groups of chickens were collected at days 0, 3, 7, 14, 28, and 42 and evaluated by ELISA using M2e synthetic peptide and M2e–HA2 fusion protein separately. Briefly, ELISA plates were directly coated with 50 μl of M2e synthetic peptide or HA stalk protein or M2e–HA2 recombinant protein (1.25 μg/ml) in carbonate bicarbonate coating buffer overnight at 4°C. Next day, plates were washed with PBS containing 0.05% Tween 20 (PBST) and blocked with 5% non-fat dry milk powder (5% NFDM in PBST) for 1 h at 37°C. Then, the antigen-coated plates were washed thrice with PBST and incubated with 50 μl of 1:50 diluted (1% NFDM in PBST) serum samples for 1 h at 37°C. After washing with PBST thrice, plates were incubated with 50 μl of 1:25,000 diluted anti-chicken immunoglobulin HRPO conjugates (Sigma-Aldrich, USA) for 1 h at 37°C. The substrate reaction was developed by adding 50 μl of 3,3′,5,5′-tetramethylbenzidine (TMB; Sigma, USA) to each well, and the reaction was stopped after 10 min with 0.4 M H_2_SO_4_. The optical density (OD) of each well was read at 450 nm in a TriStar2S LB 942 multimode reader (BERTHOLD Technologies, Germany).

### Challenge Study

Challenge experiment was carried out in the Class III Biosafety cabinets (Isolators) inside the BSL3 animal bio-containment facility of ICAR- NIHSAD, India. The immunized chickens of groups A, B, and C (except non-vaccinated and non-infected control birds) were transferred to respective isolators A, B, and C, and all were challenged intranasally with 10^8.0^ EID_50_/0.1 ml of clade 2.3.2.1 H5N1 [A/chicken/India/CA0302/2011 (H5N1)] virus and monitored continuously. Peripheral blood, oral swabs, and cloacal swabs were collected after 24 h of virus infection. PBMCs were separated from the whole blood and processed for RNA extraction. All the swabs were processed immediately and stored at −80°C until use. All the samples of challenged birds were processed following strict biosafety norms of ICAR-NIHSAD.

### Post-challenge Inflammatory Cytokine mRNA Expression Analysis

Approximately 5 × 10^6^ cells pelleted after centrifuging at 250 × g for 10 min were used for RNA extraction using an RNeasy Mini kit (Qiagen, Hilden, Germany) according to the manufacturer's protocol. The RNA was quantified using a Qubit fluorometer (Invitrogen, USA), and cDNA synthesis was carried out in a 20-μl volume using a First Strand cDNA Synthesis Kit (Fermentas, USA) with random hexamer primers from 1 μg of RNA according to the manufacturer's guidelines.The post-challenge mRNA expressions of chicken inflammatory cytokines (TGF-β, TNF-α, IFN-α, IFN-β, IFN-γ, IL-1β, IL-6, IL-4, and IL-10) and TLRs (1, 2, 3, 4, 5, 7, 15, and 21) were quantified similar to pre-challenge study.

### Quantification of Viral Load

Challenge virus shedding *via* oropharyngeal and cloacal route was measured by quantitative Real-Time RT-qPCR targeting Matrix (M) gene of AIV using M gene-specific primers, [(F) 5′- TGA TCT TCTTGA AAA TTT GCA G-3′; (R) 5′-CCG TAG MAG GCC CTC TTT TCA-3′] and probe (TTG TGG ATT CTT GAT GC) (33). Viral RNA was extracted from the swabs using QIAmp viral RNA mini kit (Qiagen) as per the manufacturer's protocol, and RT-qPCR was performed using SuperScript One-Step RT-qPCR kit (Invitrogen) using Roche 480 (Roche, USA) real-time cycler. The assay was performed in a total volume of 25 μl containing 12.5 μl of 2× master mix, 0.5 μl of Rox dye, 0.5 μl of Taq mix, 0.5 μl of forward and reverse primers (20 pmol), 0.5 μl of probe (10 pmol), 2.0 μl template RNA, and 8.5 μl of nuclease-free water to make a final volume of 25 μl. Positive and negative controls, no probe control were included in each assay. The cycling condition was as follows: one cycle of 50°C for 45 min and 95°C for 10 min, followed by 40 cycles at 95°C for 15 s and 60°C for 60 s with fluorescence acquisition. The results were determined based on the Ct values, and the copy number was calculated using standard curve for influenza M gene.

### Statistical Analysis

One-way ANOVA followed by Tukey's *post-hoc* analysis was used to compare multiple groups using SPSS 16.0 software. A *p*-value of < 0.05 was considered to indicate a statistically significant difference between groups.

## Results

### Identification of M2e and HA2 Conserved Region of Indian Avian Influenza H5N1 Viruses and Synthesis of Fusion Chimeric Protein

Based on the analysis of Indian H5N1 sequences and published data, the identified M2e region was conserved between all the clades of Indian AIV isolates except at amino acid positions 10, 11, 16, and 20. The identified M2e peptide was synthesized commercially (Genscript, NJ, USA) using Fmoc chemistry of solid phase method. The purity of the peptides was ensured by its high-performance liquid chromatography (HPLC) purification report and dissolved in water (14 mg/ml). Similarly, we have analyzed the HA2 region of different clades of Indian H5N1 virus and selected two conserved regions, namely, N-terminal HA2 (1–38) and LAH (long α-helix) HA2 (76–130) in which earlier was conserved 100% and later was conserved 95% except at amino acid positions 116, 126, and 127, respectively. Synthetic gene construct in pET32b(+) coding for M2e-HA2 was transformed into the host, E. coli Rosetta Blue (*DE3*)*pLysS*. The addition of IPTG induced the overexpression of ~38-kDa molecular weight recombinant protein, which was confirmed by Western blotting with anti-His and anti-AIV antibody (Figure 1). The expressed protein was purified by affinity chromatography using His-Bind purification kit and quantified by Qubit® fluorometer (Invitrogen, USA). The purified, pooled protein concentration was found to be 5 mg/ml and stored at −80°C. ELISA with known positive and negative AIV serum revealed that M2e–HA2 recombinant protein was highly reactive with positive serum (OD@450 nm >0.50) and failed to react with negative control (SPF chicken) serum (OD@450 nm <0.20) (Figure 1D).

### Kinetics of CD4^+^and CD8^+^ Population

**Figure 2:**
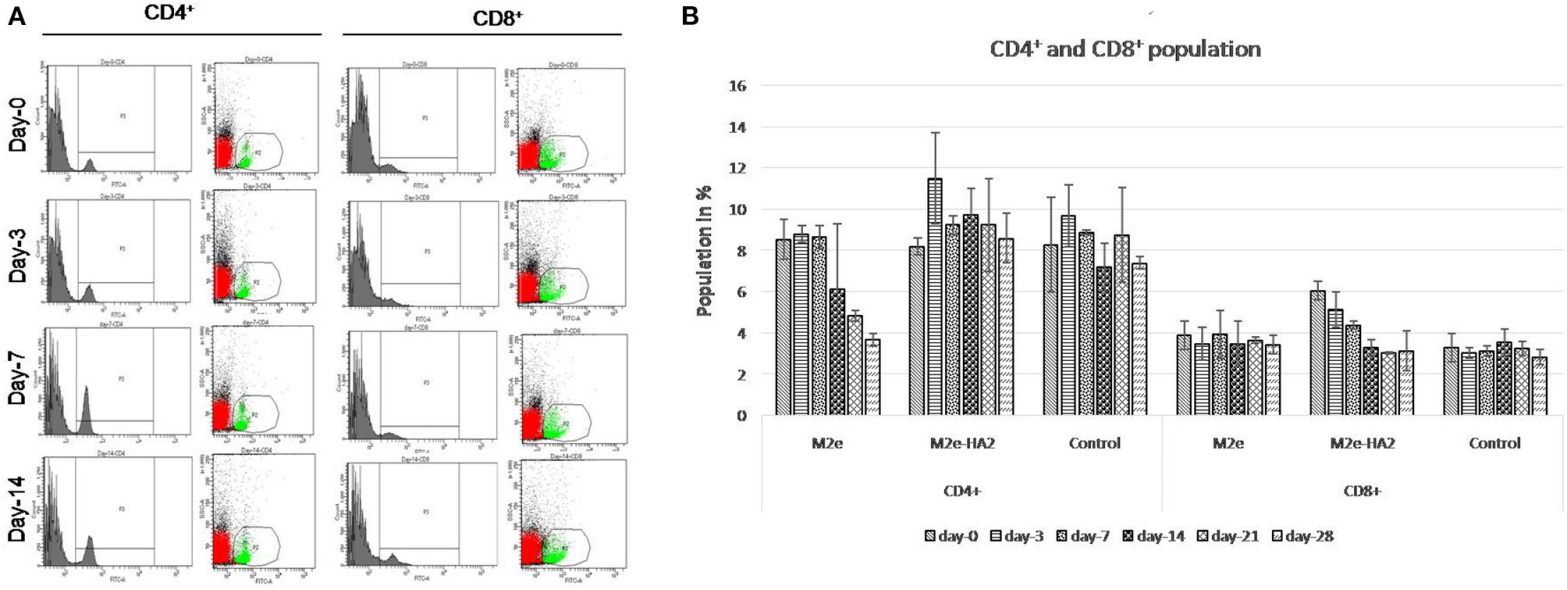
Kinetics of immune cells (CD4^+^ and CD8^+^) proportion (mean ± SE) following immunization with M2e, M2e–HA2, and control groups quantified by flow cytometry at different day intervals **(A,B)**. CD4^+^ population in the M2e–HA2 group was higher than that in the control as well as M2e group during the study period and maximum on 3DPV. Data are represented as mean ± SD (*n* = 6) and analyzed by ANOVA, and no significant difference was observed between groups at the same interval.

CD4^+^ population in M2e–HA2 was higher than that in control as well as M2e group at all-time interval, and the maximum elevation was at 3 days post-immunization (not significant). However, M2e group showed the declining of CD8^+^ population from day 14 and was lower than that even in the control group. The percentage of CD8^+^ population in the M2e–HA2 group was gradually decreased from day 0, whereas there was no significant difference observed between control and M2e group (Figure 2).

### M2e–HA2 Recombinant Fusion Protein Induced Lymphocyte Proliferation Following Immunization

**Figure 3:**
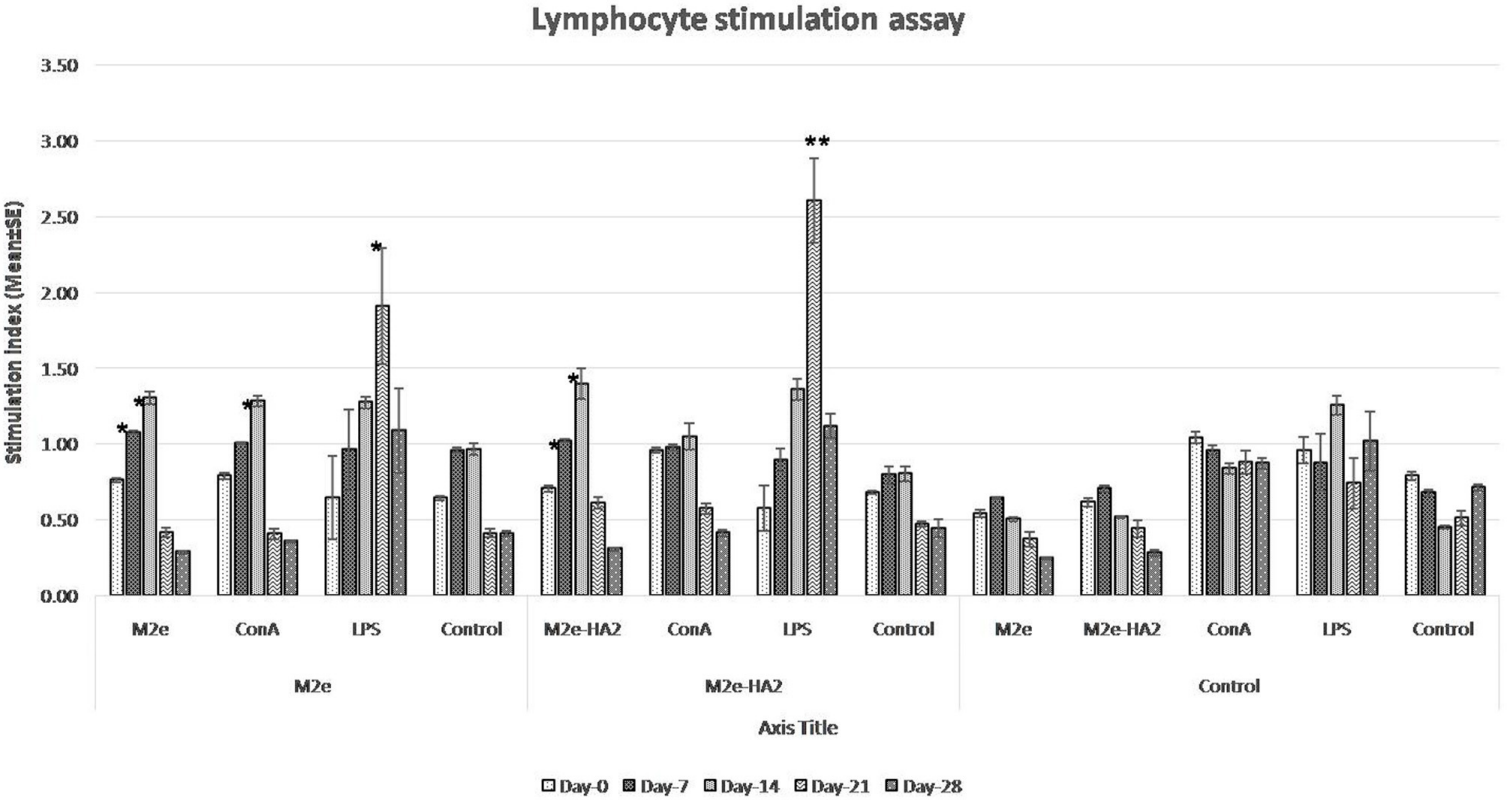
Lymphocyte proliferation assay of M2e- and M2e–HA2-immunized and non-immunized control chickens. Stimulation index (SI) was increased gradually from day 0 to day 14 in the M2e and M2e–HA2 groups. SI by lipopolysaccharide (LPS) with all the groups was maximum and higher than concanavalin A (ConA) from day 14 and reached the peak at day 21 DPV. Data are means ± SD (*n* = 6), statistical significance (*p ≤ 0.05, **0.01) vs. non-immunized control groups in the same time interval. The control mentioned within group is the unstimulated lymphocytes of the same group.

Lymphocyte proliferation assay revealed that SI was increased gradually from day 0 to day 14 in the immunized group (Figure 3). However, the maximum fold increases in M2e and M2e–HA2 groups were 1.36 and 1.48 with their concerned antigen, respectively. At the same time, SI by LPS with all the groups was maximum and higher than that by ConA from day 14 and reached peak at day 21. Maximum SI by LPS was noticed with the M2e–HA2 group (2.61 ± 0.28-fold) followed by M2e (1.91 ± 0.38-fold), which was higher than that of the control group (1.26 ± 0.07-fold). ConA- and LPS-mediated SIs (1.04–1.26) were almost equal in the control group. Day-wise comparison revealed that SI was significantly increased from day 0 to day 14 in all immunized groups (*p* < 0.05).

### Elevated Level of Pro-inflammatory Cytokine and Toll-Like Receptor mRNA Expression

**Figure 4:**
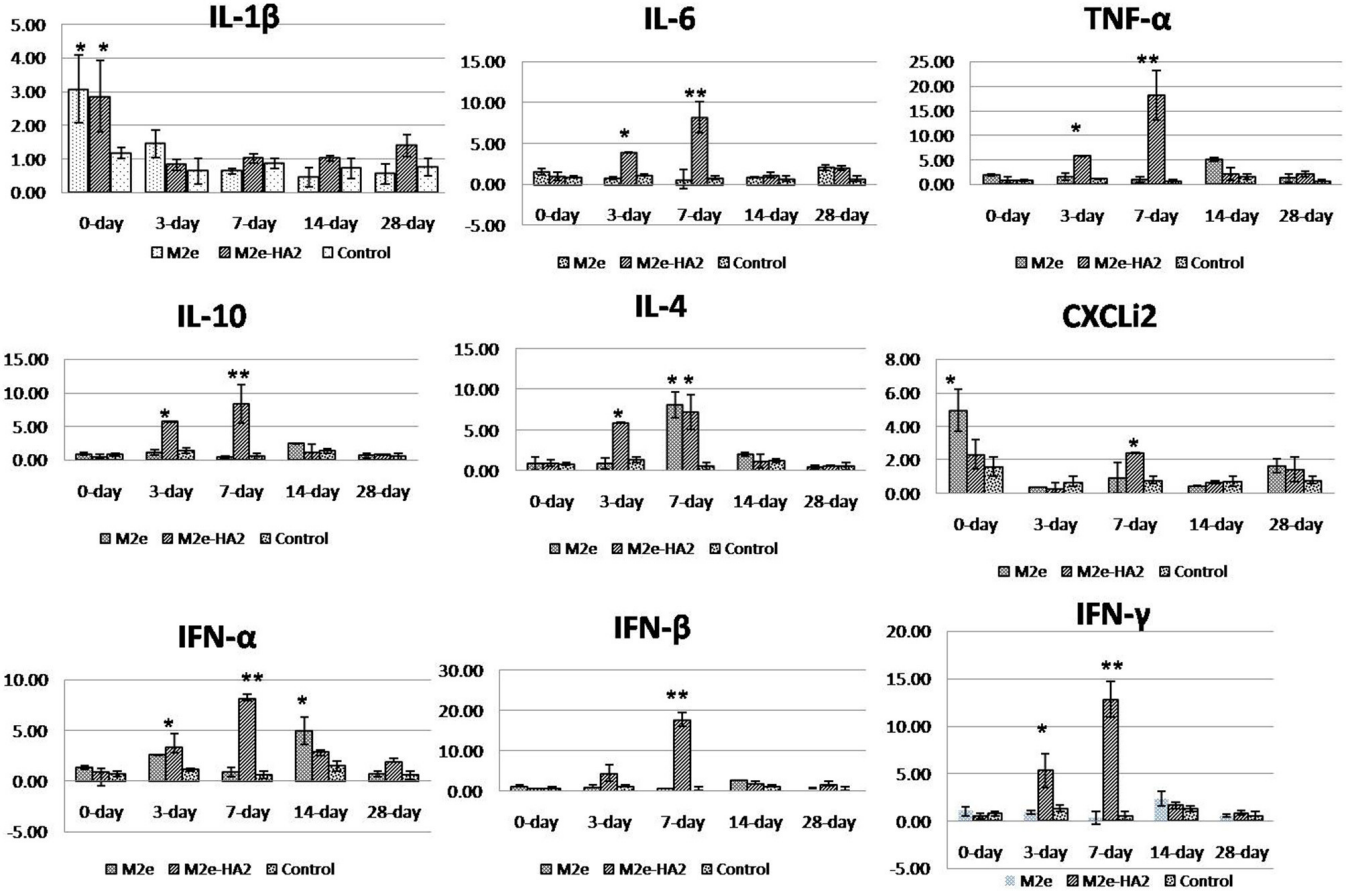
Comparison of relative inflammatory cytokine mRNA expressions in M2e, M2e–HA2, and control chicken peripheral blood mononuclear cells (PBMCs). M2e–HA2-immunized chickens showed upregulation of interleukin (IL)-6, tumor necrosis factor (TNF)-α, chemokine CXCLi2, interferons (IFNs) (α, β, and γ) on 3 and 7 days post-primary immunization than M2e and control group. Data are means ± SD (*n* = 6), statistical significance (*p ≤ 0.05, **0.01) vs. control non-immunized groups in the same time interval.

Analysis of pro-inflammatory cytokines revealed that IL-1β mRNA expression was higher in the initial period (*p* < 0.05) followed by a gradual decrease in all the groups including control (Figure 4). Expression of IL-6 mRNA was not different in M2e and control, whereas it was gradually increased from day 0 to day 7 (eight-fold) in the M2e–HA2 group (*p* < 0.01) and then started to decline. TNF- α (LITAF) mRNA expression was maximum at day 7 of the M2e–HA2 group (18-fold) (*p* < 0.01), which was all time point higher than that of the other groups. Analysis of anti-inflammatory cytokine IL-10 and TGF-β revealed that there was no difference in the expression of TGF-β mRNA, whereas IL-10 mRNA expression was maximum (six- and eight-fold) in the M2e–HA2 group at days 3 and 7 (*p* < 0.01), followed by that in the M2e group. Analysis of IFN mRNA revealed that IFN-α was maximum (eight-fold) at day 7 of the M2e–HA2 group followed by day 14 of M2e. Regarding IFN-β and IFN-γ mRNA expression, they were maximum (18-fold and 13-fold) at the seventh day followed by the third day (4.8- and 5.3-fold), respectively, in the M2e–HA2 group (*p* < 0.01), then by that in the M2e group. Overall, all the three IFN mRNAs were expressed higher in the M2e-HA2 group followed by that of the M2e group then that of the control group at days 3 and 7 (*p* < 0.05).

**Figure 5:**
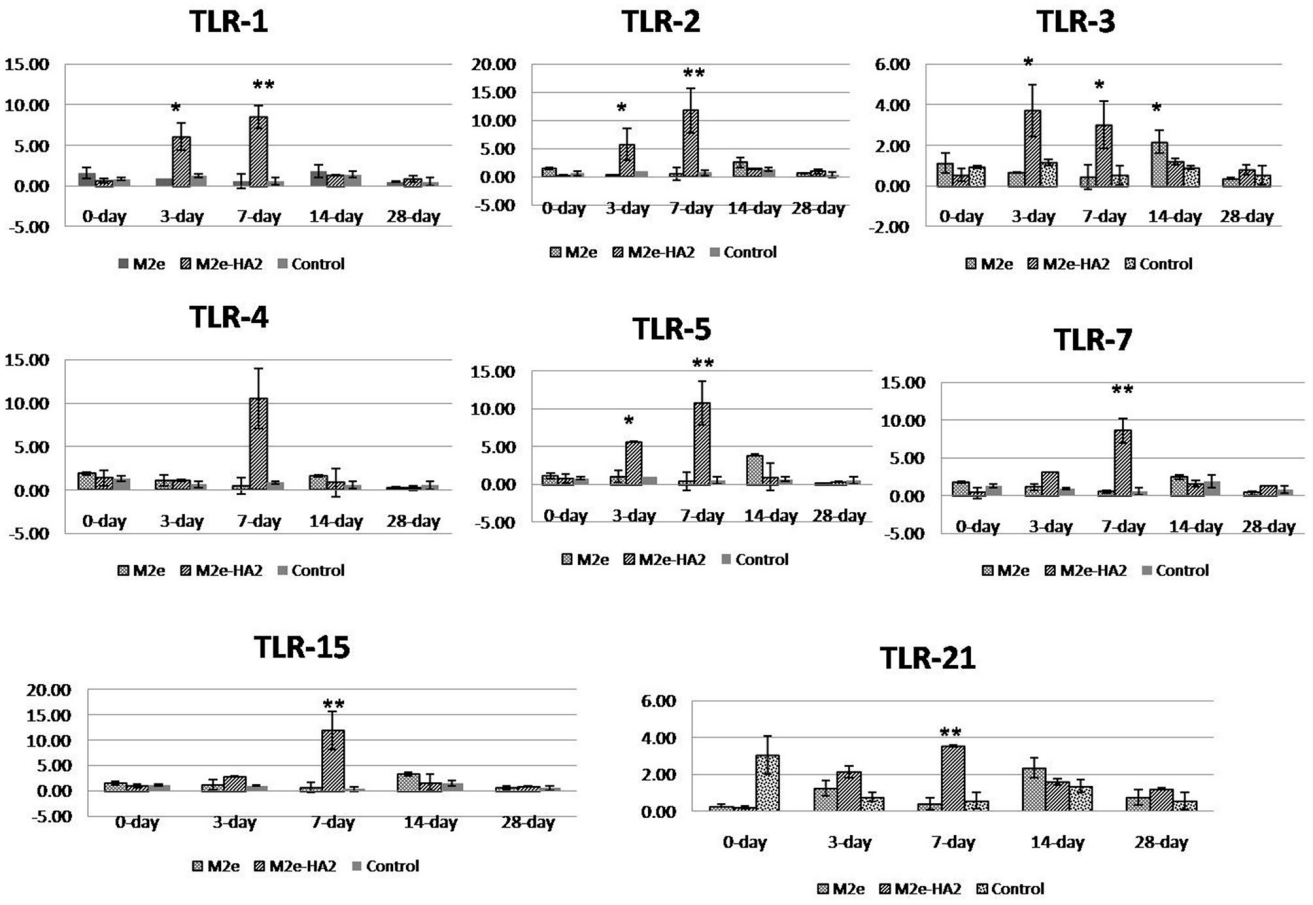
Comparison of relative expression of Toll-like receptor (TLR) mRNAs in M2e- and M2e–HA2-immunized and non-immunized control chicken peripheral blood mononuclear cells (PBMCs). M2e–HA2-immunized chickens showed upregulation of all the TLR mRNAs than M2e and control on 3 and 7 days post-immunization. Data are means ± SD (*n* = 6), statistical significance (*p ≤ 0.05, **0.01) vs. control non-immunized groups in the same time interval.

All the chicken TLR mRNA expressions were analyzed by RT-qPCR. Expression of TLR1, TLR2, TLR3, TLR4, TLR5, TLR7, and TLR21 mRNA was maximum at days 3 (*p* < 0.05) and 7 (*p* < 0.01) of post-immunization in the M2e–HA2 group followed by day 14 of the M2e group (Figure 5). However, TLR 15 mRNA expression was maximum in day 7 of M2e–HA2 (12-fold) (*p* < 0.01) followed by day 14 of the M2e group (3.5-fold). No change in TLR mRNA expression was observed in the control group.

### Detection of M2e and HA2-Specific Antibody by ELISA

**Figure 6:**
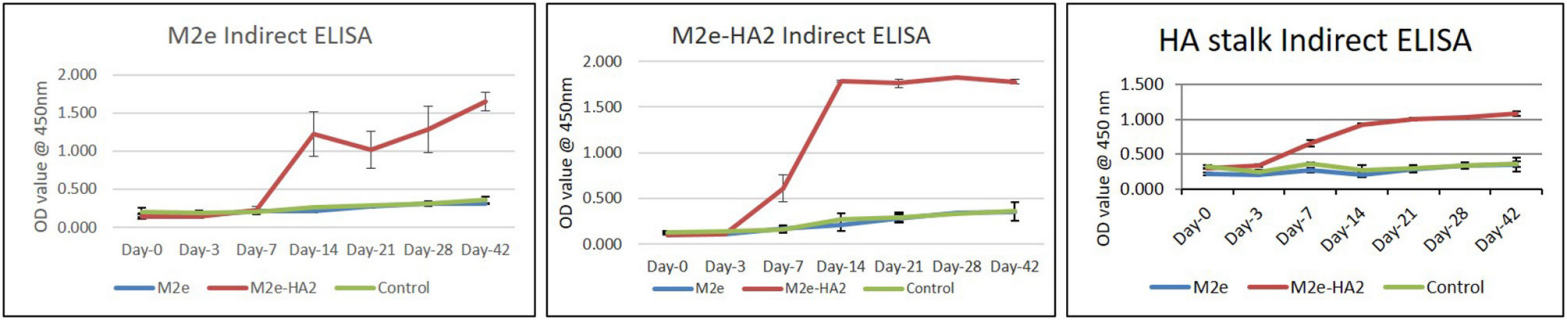
M2e synthetic peptide, recombinant M2e–HA2 protein, and recombinant HA stalk protein-based indirect ELISA of M2e- and M2e–HA2-immunized chicken sera of different day intervals along with control group. Data are means ± SD (*n* = 6).

Clades 2.2 and 2.3.2.1 of AIV hyper immune chicken sera were reacted well with M2e–HA2 recombinant protein and less efficiently with M2e synthetic peptide in indirect ELISA, with an average OD_450_ value up to 2.5 and 0.5, respectively, at 1:200 dilutions of serum. ELISA using M2e–HA2 recombinant protein as coating antigen revealed that the M2e–HA2 recombinant fusion protein has induced M2e–HA2-specific antibody from 7 days post-primary immunization (Figure 6) and then the titer gradually increased after the booster dose. At the same time, M2e peptide ELISA was also carried out with the same serum to differentiate M2e-specific antibody. M2e peptide ELISA revealed that M2e-specific antibody elicitation started from day 14 and gradually increased further after the booster. M2e-specific differential ELISA revealed that the production of HA2-specific antibody was earlier (at day 7) than M2e-specific antibody (day 14). The early elicitation of HA2-specific antibody was also confirmed by recombinant HA stalk protein-based indirect ELISA (Figure 6C). In contrast, only background level of antibody responses was detected in the chicken immunized with synthetic peptides M2e or control group in both the ELISA.

### Drastic Reduction of Pro-inflammatory Cytokine Genes in Avian Influenza Virus-Challenged Chickens by M2e–HA2

**Figure 7:**
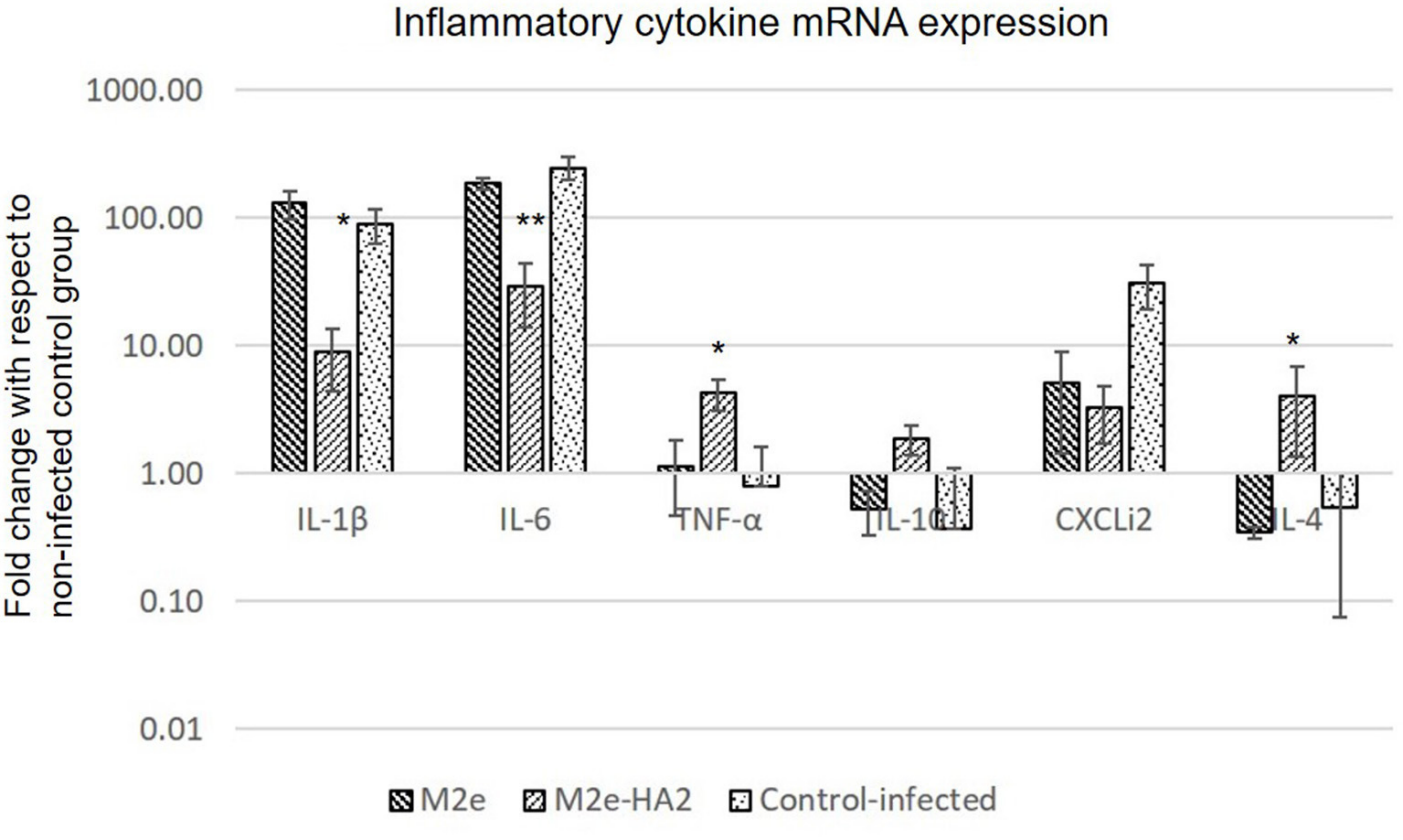
Comparison of relative expression of inflammatory cytokines mRNA in M2e, M2e–HA2, and control chicken peripheral blood mononuclear cells (PBMCs) followed by challenge with clade 2.3.2.1 H5N1. M2e–HA2-immunized chickens showed drastic reduction of interleukin (IL)-1β and IL-6 than M2e and control. Data are means ± SD (*n* = 6), statistical significance (*p ≤ 0.05, **0.01) vs. control-infected groups in the same time interval.

Pro-inflammatory cytokine genes (IL-1β, IL-6, and CXCLi2) were highly upregulated in the H5N1 HPAI-infected control group, whereas the same were drastically reduced in the M2e–HA2-immunized group [*p* ≤ 0.01 for IL-16 (900-fold); *p* ≤ 0.05 for IL-1β (30-fold) and CXCLi2 (130-fold)]. Reduction of IL-6 and CXCLi2 was noticed in the M2e group also with lesser percentage than that in the M2e–HA2 group. At the same time, TNF-α (LITAF), IL-4, and IL-10 were slightly downregulated in the infected control group, whereas significant upregulation was noticed in the M2e–HA2 group (Figure 7).

### Induction of CD4^+^ and CD8^+^ Population Depletion by Avian Influenza Virus

**Figure 8:**
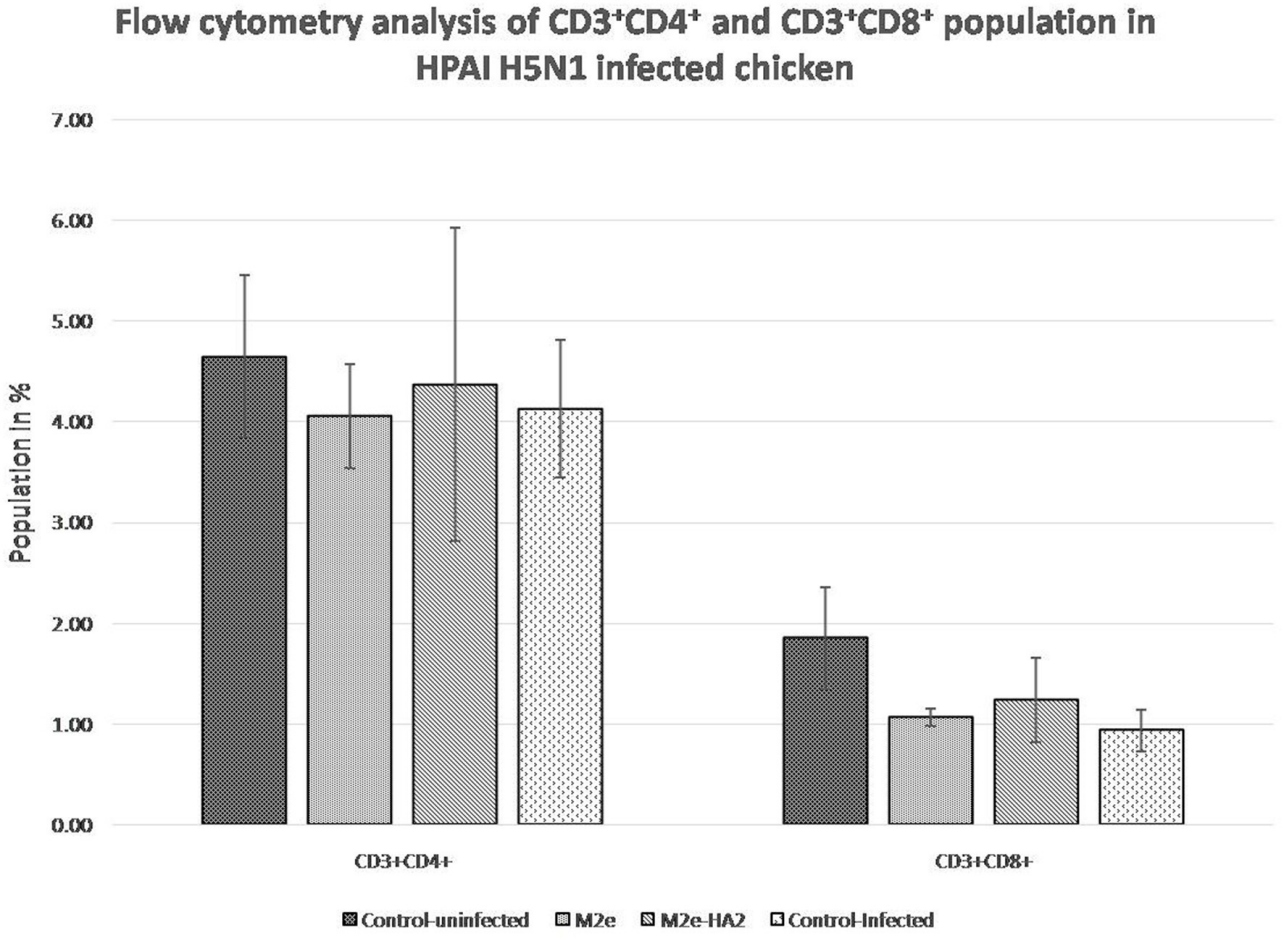
Comparison of CD4^+^ and CD8^+^population in M2e- and M2e–HA2-immunized and control chickens followed by challenge with clade 2.3.2.1 H5N1. Data are means ± SD (*n* = 6), statistical significance (*p ≤ 0.05, **0.01) vs. control non-infected group.

FACS analysis of CD3^+^CD4^+^ and CD3^+^CD8^+^ cell population after 24 h of virus infection revealed that H5N1 HPAI induced the depletion of both populations (Figure 8). The M2e–HA2 group showed slight inhibition of depletion of both populations but not up to the level of the uninfected control group. However, inhibition of depletion by M2e–HA2 was comparatively higher than that in the M2e peptide group.

### Upregulation of Toll-Like Receptor Gene Expression

**Figure 9:**
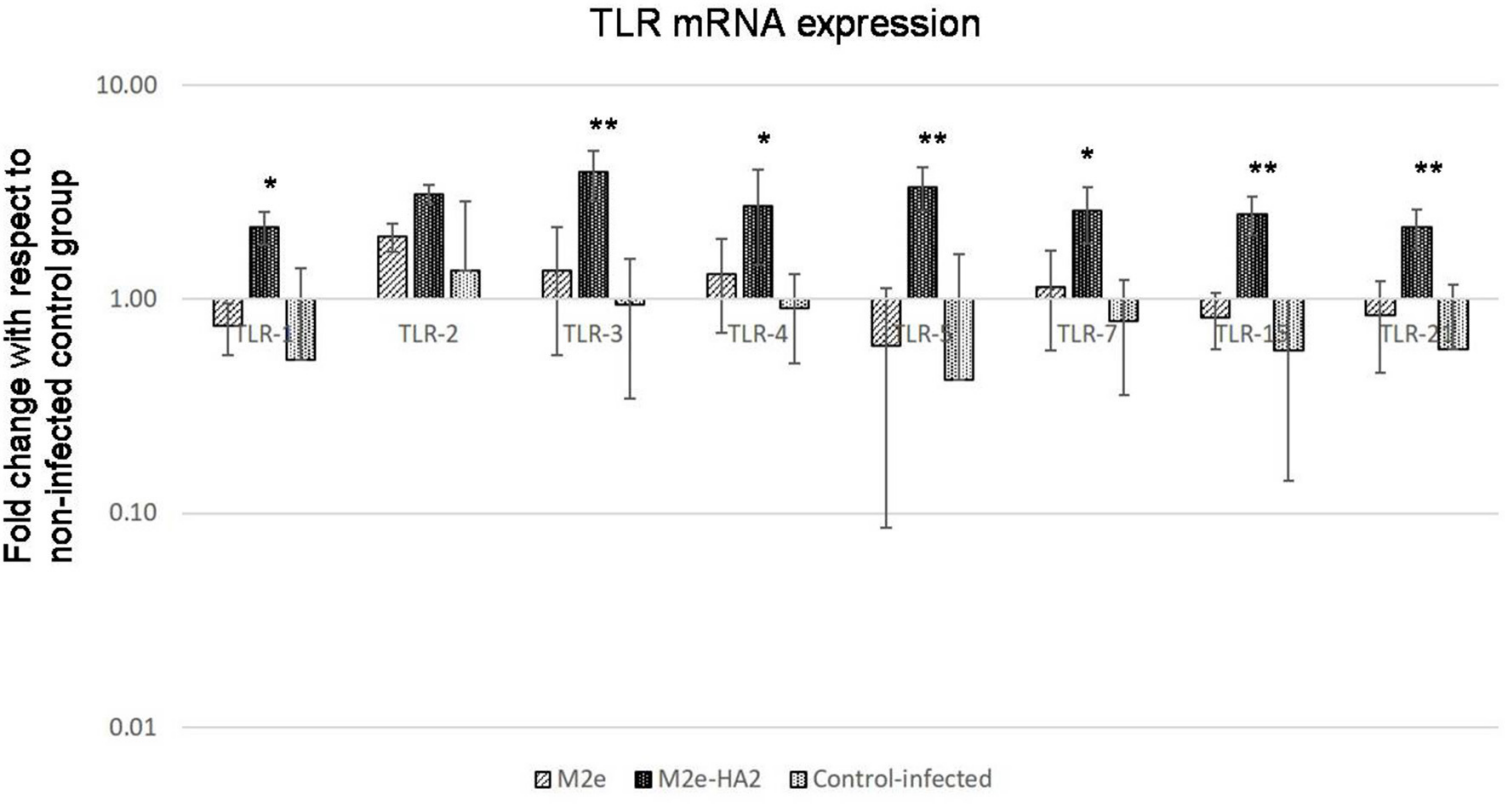
Comparison of Toll-like receptor (TLR) mRNA expressions in M2e- and M2e–HA2-immunized and control chicken peripheral blood mononuclear cells (PBMCs) followed by challenge with clade 2.3.2.1 H5N1. M2e–HA2-immunized chickens showed upregulation of all the TLR mRNAs than M2e and control-infected. Data are means ± SD (*n* = 6), statistical significance (*p ≤ 0.05, **0.01) vs. control-infected groups in the same time interval.

Most of the TLR genes were downregulated in H5N1 HPAI-infected control group at 24 h of infection (Figure 9). At the same time, all showed significant upregulation (2–4-fold) in the M2e–HA2 group (*p* < 0.01).

### Viral Shedding in Oropharyngeal and Cloacal Swabs

**Figure 10:**
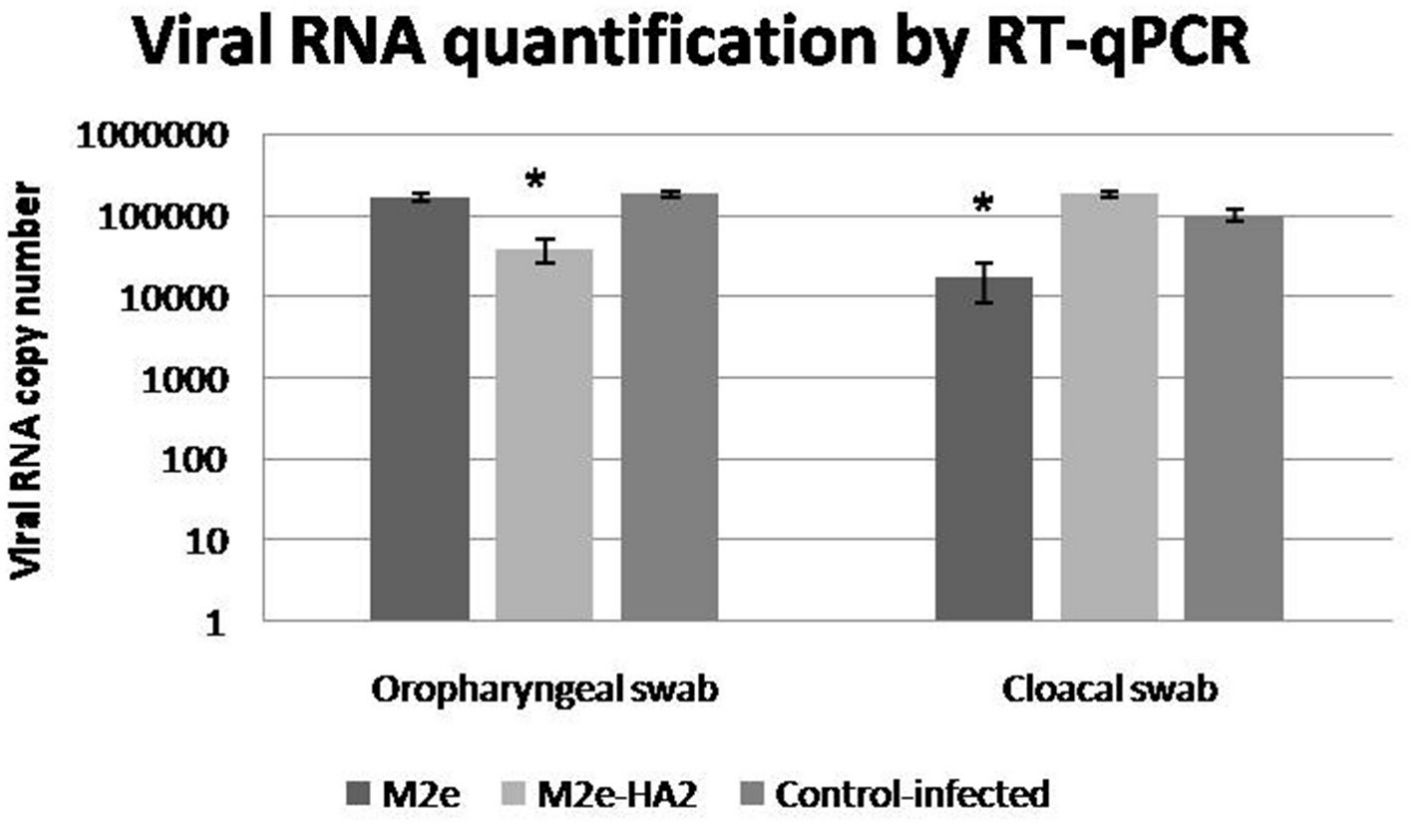
Viral shedding detected by qRT-PCR in cloacal and oropharyngeal swabs of specific pathogen-free chickens challenged with 10^8.0^ EID_50_/0.1 ml of clade 2.3.2.1 H5N1. Control-infected birds shed more virus in their oropharyngeal swabs followed by M2e and M2e–HA2 groups after 24 h. Data are means ± SD, statistical significance (*p ≤ 0.05, **0.01) vs. control-infected group.

Viral RNA shedding analysis by RT-qPCR at 24 h of infection revealed that the control-infected birds shed more virus in their oropharyngeal swabs followed by M2e and M2e–HA2 groups in descending order (Figure 10). At the same time, the M2e–HA2 group showed more viral RNA in their cloacal swab than control-infected and M2e groups. However, all the birds including M2e–HA2-immunized and -challenged died at 48 h of virus infection with typical clinical signs of AIV infection.

## Discussion

The extracellular domain of influenza M2 protein (M2e) is highly conserved among influenza A viruses and considered an appropriate target for the development of universal influenza vaccine with broad-spectrum protection (23). Analysis of representative Indian H5N1 sequences of 2006-15 revealed the high conservation between them except at amino acid positions 10, 11, 16, and 20 of M2e. Earlier reports also reveal that M2e residues are variable between 10 and 24 but showed conservation of Arg12, Trp15, Cys17, Cys19, and Ser22, suggesting that these residues in M2e are functionally important (34), and the same conservation is noticed in Indian isolates. In earlier studies, protective M2e antibodies had been induced in a variety of ways including full-length protein with adjuvant (35), DNA administration (36), fusion to hepatitis B core protein (37, 38), keyhole limpet hemocyanin (39, 40), flagellin (41), as liposomes (42), using viral vectors (43–47), tandem repeat formats (M2e–MAP) (48, 49), VLPs (23, 50), recombinant expression with CD154 epitopes (51), and chitosan nanoparticle encapsulation (52). The HA2 subunit (221 amino acids) structure is composed of two anti-parallel α-helixes and is more conserved than HA1 (53). Analysis of HA2 sequences revealed that the N-terminal region 1–38 is completely conserved among all isolates and reported to provide intra-subtype cross-protection in mice (54). Similarly, analysis of LAH (76–130 AA) revealed 95% of conservation between the Indian sequences and also reported to elicit neutralizing antibodies and efficacious protection against H3 and moderate protection against other subtypes H5, H7, H2, and H1 in mice (55).

In this study, a novel approach was attempted by making a synthetic construct to link M2e with another conserved region of AIV, HA2 to facilitate the formation and maintenance of the larger immunogenic molecule for improving the immunogenicity of M2e (56). Combining of HA and M2e is an attractive approach for the development of broad-spectrum universal influenza vaccines, and the same had been reported by earlier workers (34, 52). Instead of selecting whole HA region, here, we have selected two immunogenic subtypic cross-protective regions, namely, HA2 (1–38) and LAH HA2 (76–130) as reported earlier (54, 55). It is expected that insertion of few conserved epitopes into recombinant proteins in any universal vaccine will lead to enhanced protective efficacy (57), hence the second subunit (HA2) of the conserved antigen was selected. In previous reports, it has been shown that HA2 (aa76–130)-based synthetic peptide vaccine using HA from A/Hong Kong/1/1968 (H3N2) provides protection in mice against divergent subtypes H3N2, H1N1, and H5N1 (58). Therefore, we used a conserved fragment of HA2 (76–130) along with HA2 fusion peptide (1–38AA) as a second target antigen for the design of recombinant protein with broad-spectrum protection. Thus, the gene construct was designed to produce M2e–HA2 fusion recombinant protein with linkers in between them.

Cellular immune response was monitored after immunization by lymphocyte proliferation assay, flow cytometry, and cytokine mRNA analysis. Lymphocyte proliferation assay revealed that SI was increased gradually from day 0 to day 14 in the immunized group with their concerned antigen and up to 21 days with LPS than in the control group. Maximum SI with LPS at 14 and 21 days ensures the stimulation of B cells in immunized groups, which also supported by non-stimulant response of the control group. Upregulated expression of IL-10 and TNF-α mRNA expression in M2e-HA2 from 7 days onward also support the induction of humoral immunity because IL-10 and TNF-α are the important cytokines for immunoglobulin class switching, an important phenomenon in humoral immunity (59–61). Increased level of IL-4 mRNA from day 7 of the M2e–HA2 group also suggests the conversion of Th2-mediated humoral immunity (62, 63). Increased percentage of CD4^+^ cells in the M2e-HA2 group than control and M2e indicated the enhanced cellular immune response (64).

In chickens, the currently known TLRs are TLR-1 LA, TLR-1 LB, TLR-2, TLR-3, TLR-4, TLR-5, TLR-7, TLR-15, and TLR-21. TLR-3 and TLR-7 recognize dsRNA and ssRNA molecules, respectively, in the host cells (65, 66). The chicken TLR-21 is a functional homolog of mammalian TLR-9, which induces NFκ-B production after stimulation with deoxyoligonucleotides containing CpG motifs (67). All the TLRs have been upregulated in the M2e–HA2 group at day 3 and day 7 to maximum level than those in the M2e and control groups, and the same were maximum in the M2e group on the 14th day than those in the M2e-HA2 and control groups. The results of our study indicate that conjugating M2e with HA2 effectively mediates early upregulation of TLRs than M2e alone, thereby enhancing the innate and adaptive immunity because the TLRs after stimulation by their ligands follow the cascade events of pro-inflammatory cytokine production and upregulation of co-stimulatory molecule expression, subsequently initiating adaptive immunity (68).

Although M2e-mediated humoral immunity against influenza virus has been reported in earlier studies (34, 69), most of these vaccine studies on M2 were performed in mice, while few experiments had been described for chicken with variable outcome (51, 52, 57, 70). M2e is generally a weak antigen (71)—a fact thought to be largely due to its low abundance compared with other proteins. Thus, it was hypothesized that a robust humoral immune response would be induced against M2e by linking with another conserved region of AIV, HA2, so that we could elicit immune response to two conserved regions of AIV at a time for effective response.

In this study, we also evaluated the humoral immune response of M2e–HA2 fusion recombinant protein and M2e synthetic peptide in chicken. Sera obtained from immunized chicken of all groups were found to be HI negative. Then, all the sera were tested by M2e peptide ELISA and found that the group vaccinated with M2e–HA2 fusion protein showed a positive reaction, whereas M2e alone and control group failed to produce antibody against M2e. Same types of approach were followed by earlier workers (53). The OD_450_ value of hyper immune sera against M2e–HA2 was comparatively higher than that of M2e peptide alone, indicating the abundance of HA2 antibody in natural infection than M2e. Also, we have noticed that immunization with M2e–HA2 fusion recombinant protein has induced M2e-specific antibody from day 14 of immunization whereas HA2-specific antibody was detected from day 7 of immunization and were detected by M2e peptide and M2e–HA2 recombinant protein ELISA, respectively. This observation suggests that the M2e–HA2 recombinant fusion protein elicited HA2-specific humoral immunity earlier than M2e, but at the same time, M2e-specific antibody also elicited in good amount but not earlier than HA2, and this may be due to high immunogenicity and larger molecule nature of HA2. At the same time, M2e peptide monomer was inefficient to produce an antibody response, and the same type of observation has been reported by Swinkels et al. (72), whereas immunization with M2e peptide tetrameric construct showed a significant antibody response after the booster. To the best of our knowledge, this is the first attempt to construct a fusion protein with two truncated, conserved immunogenic subunits of HA2 along with M2e to elicit a broad immune response in chicken against M2e and HA2 regions of AIV.

Recent study suggests that the host pro-inflammatory responses are one of the major contributing factors in the pathogenesis of H5N1 HPAI virus infection in chicken, and the fatal outcome could be mediated by a cytokine storm or hyper-acute dysregulation of pro-inflammatory cytokines similar to human H5N1 HPAI virus infection (73). Similar hyper-acute dysregulation of pro-inflammatory cytokines has been observed in our study also in control-infected group, whereas the cytokine response was drastically reduced in the M2e–HA2 group as a protective response. Although the cross-protective properties of M2e-based vaccines and the role of anti-M2e antibodies in cross-protection against influenza A viruses have been shown by a number of studies (41, 74–77), this M2e–HA2 fusion protein has failed to protect the chicken from a high dose (10^8.0^ EID_50_/0.1 ml) of H5N1 HPAI challenge after 48 h even after eliciting the antibodies to its conserved antigens (M2e and HA2) and inhibited the depletion of CD4^+^ and CD8^+^ cells to a certain extent, which is an essentiality for the novel vaccines (78–80).

In this study, we have observed that the M2e alone as a synthetic peptide was not able to induce an antibody response, whereas M2e–HA2 recombinant protein has induced antibody against both M2e as well as HA2. M2e-HA2 recombinant protein has drastically reduced the pro-inflammatory cytokines and upregulated innate immune system of chicken but failed to protect from a higher dose of HPAIV H5N1 challenge. Findings of this study indicate that despite the conservation, merely M2e and HA2-mediated immune response alone may be insufficient to protect chicken from HPAI H5N1 virus challenge, and this will be very useful in future development of universal influenza vaccine targeting M2e and HA2 especially for chicken.

## Data Availability Statement

The original contributions generated in the study are included in the article/Supplementary Material, further inquiries can be directed to the corresponding author.

## Ethics Statement

The animal study was reviewed and approved by Institute Animal Ethics Committee (IAEC) of ICAR-National Institute of High Security Animal Diseases, Bhopal, India.

## Author Contributions

SK and SB designed the study. SK performed the experiments. MK, DS, BP, DD, and RS helped in the animal experiment. NM helped in the analysis. KR helped in flow cytometry analysis. SB and VS edited the manuscript. All authors contributed to the article and approved the submitted version.

## Conflict of Interest

The authors declare that the research was conducted in the absence of any commercial or financial relationships that could be construed as a potential conflict of interest.
