# Supplementary figures and images for: Elicitation of Highly Pathogenic Avian Influenza H5N1 M2e and HA2-Specific Humoral and Cell-Mediated Immune Response in Chicken Following Immunization With Recombinant M2e–HA2 Fusion Protein
Source: Front Vet Sci. 2021 Feb 5;7:571999. doi: 10.3389/fvets.2020.571999 (PMC7892607; doi:10.3389/fvets.2020.571999)

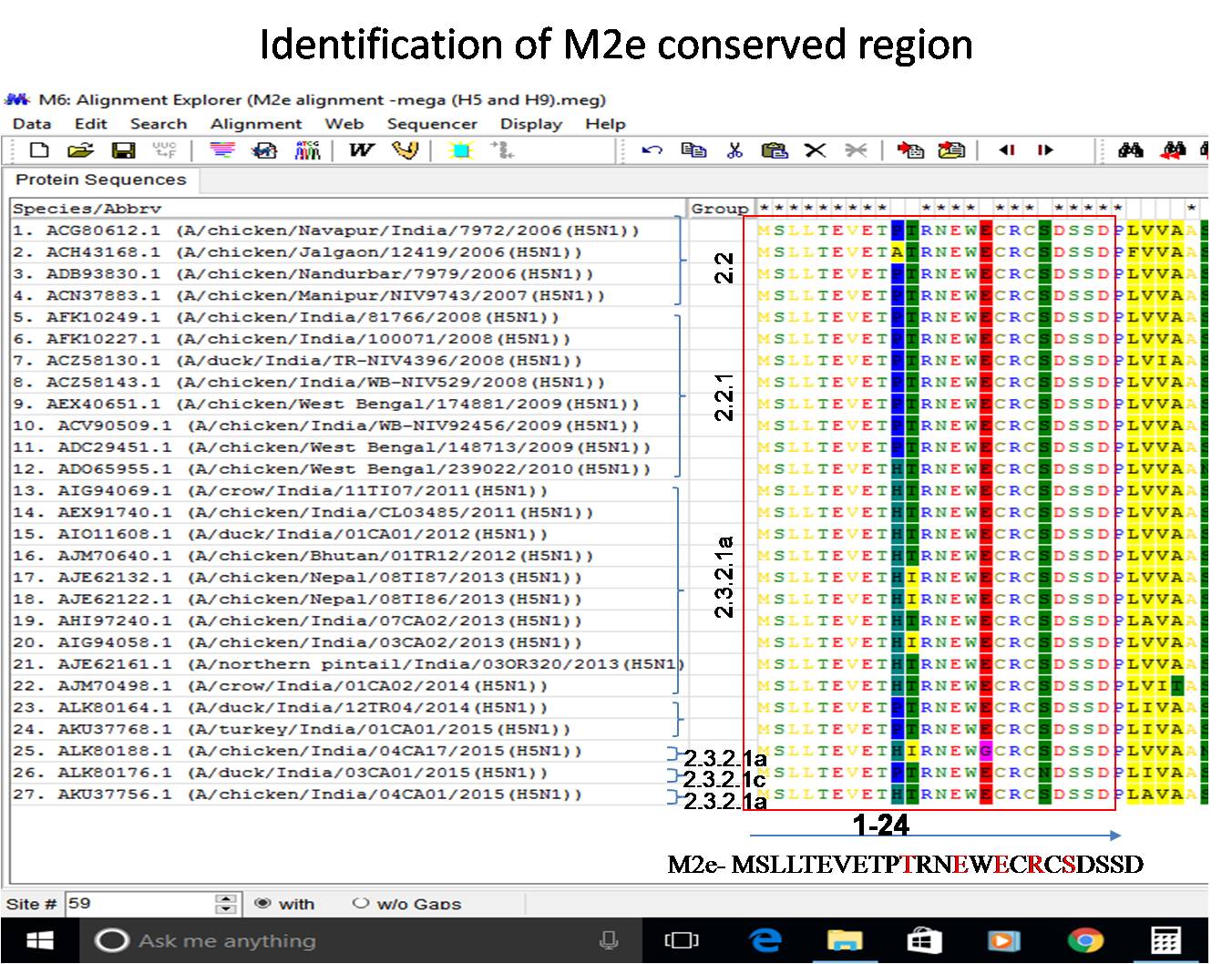

Supplement: Supplementary file 1 [file Image_1.JPEG]

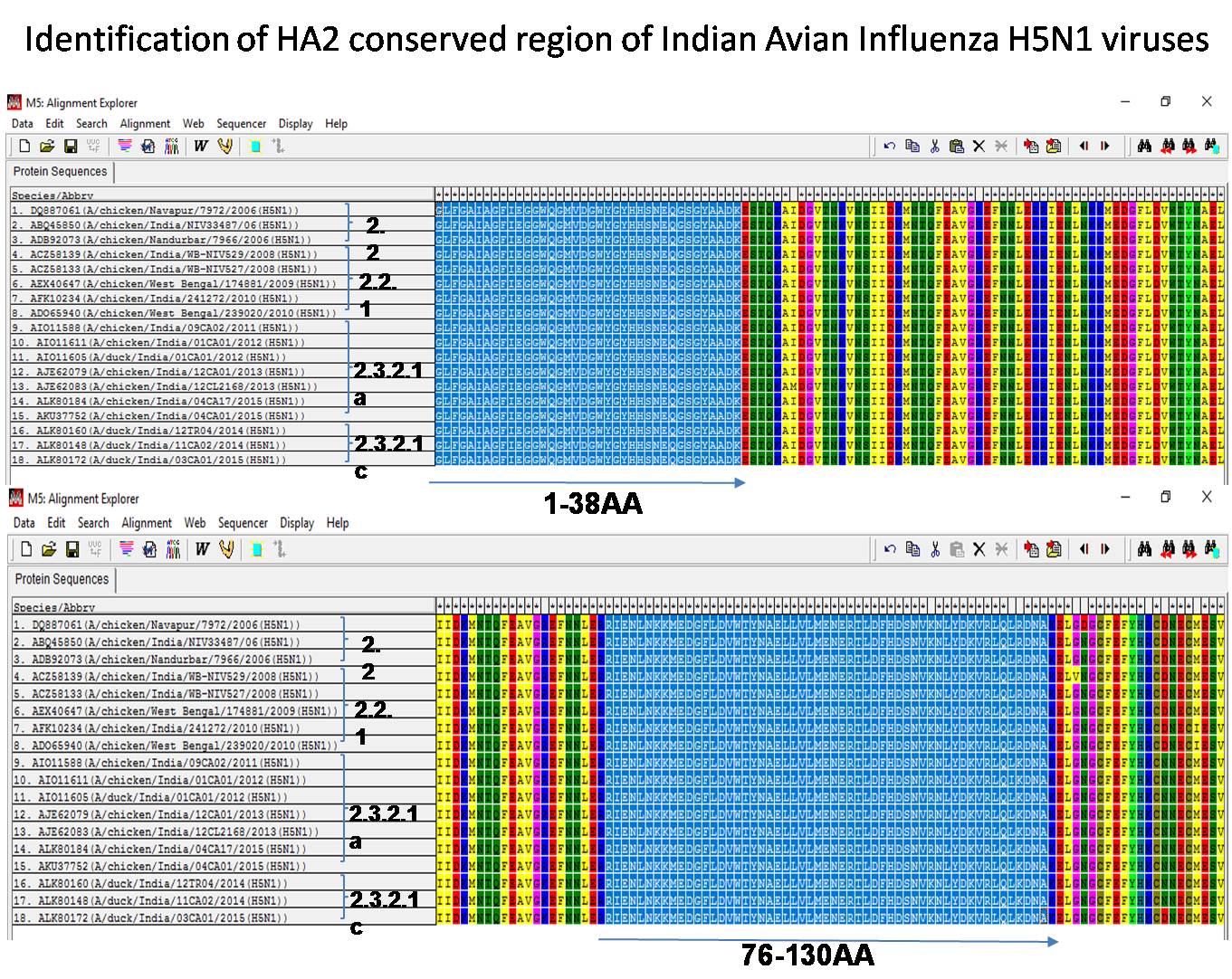

Supplement: Supplementary file 2 [file Image_2.JPEG]
