# Supplementary material for: Elicitation of Highly Pathogenic Avian Influenza H5N1 M2e and HA2-Specific Humoral and Cell-Mediated Immune Response in Chicken Following Immunization With Recombinant M2e–HA2 Fusion Protein
Source: Front Vet Sci. 2021 Feb 5;7:571999. doi: 10.3389/fvets.2020.571999 (PMC7892607; doi:10.3389/fvets.2020.571999)
Supplement: Supplementary file 3 [file Table_1.DOCX]

Table1. Evaluation of reactivity of rM2e-HA2 with chicken polyclonal sera raised against HPAI H5N1 whole viruses, H9N2, NDV and negative control by indirect ELISA.

| rM2e-HA2 antigen concentration | | |  |
| --- | --- | --- | --- |
| 2.5µg/ml | 1.25µg/ml | 0.625µg/ml | Serum dilution (1:100) |
| 2.48 | 1.14 | 0.77 | H5N1 Clade 2.2 |
| 2.3 | 1.28 | 1.02 | H5N1 Clade 2.3.2.1 |
| 1.2 | 0.36 | 0.24 | H9N2 |
| 0.4 | 0.32 | 0.17 | NDV |
| 0.42 | 0.16 | 0.15 | Negative SPF chicken serum |
